# Phenotypic identification of different β-Lactamases in intrinsic and acquired colistin resistant uropathogenic gram negative bacteria

**DOI:** 10.12669/pjms.40.6.8516

**Published:** 2024-07

**Authors:** Ambreen Arif, Ihasn Ullah, Ronaq Zaman, Arif Mehmood Khan

**Affiliations:** 1 Ambreen Arif, Health Research Institute, NIH, Islamabad, Pakistan. Khyber Medical University Peshawar, Pakistan; 2 Ihsan Ullah, Associate Professor, Khyber Medical University Peshawar, Pakistan; 3 Ronaq Zaman, Assistant Professor, Kabir Medical College, Peshawar, Pakistan; 4 Arif Mehmood Khan, FCPS Bannu Medical College, Bannu, Pakistan

**Keywords:** Multi drug resistance, Extended drug resistance, MBL, ESBLs, AmpC, Uropathogenic Gram negative bacteria

## Abstract

**Objective:**

Identification of MBL, AmpC and ESBLs in colistin intrinsic and acquired resistant uropathogenic gram negative bacteria.

**Method:**

Urine samples were collected from Hayatabad Medical Complex, Peshawar during 17 January to 30 June 2019. Collected urine samples were aseptically transported microbiology lab of Health Research Institution (HRI), National Institute of Health (NIH), Khyber Medical College, Peshawar and streaked on different media. Positive growth was identified by API-10s. Antibiotic sensitivity profile was done by Modified Kirby Bauer disc diffusion method. Detection of metallo βlactamases (MBL) production by Imipenem EDTA synergy test, Double Disc Synergy Test (DDST) for detection of ESBLs and D-test for the detection of inducible AmpC beta lactamases test was used. Colistin resistance was identified via broth micro dilution according to CLSI manual. Colistin resistant bacteria was divided in two categories; acquired and intrinsic resistant bacteria according to CLSI manual.

**Results:**

Out of 2000 urine samples, 281(14%) gram-negative bacteria were isolated. Among positive samples, acquired colistin resistant bacteria were 241 and intrinsic resistant bacteria were 40 isolates. MBL was produce by twenty one (11.7%) *E.coli* and seventeen (40.5*%) Pseudomonas aeruginosa. E. coli, Pseudomonas aeruginosa, Klebsiella Pneumoniae, Serratia Oderifora* and *Proteus Marblis* were ESBLs producing bacteria. AmpC production was prevalent in fourteen (7.8%) *E. coli* and twelve (28.6*%) Pseudomonas aeruginosa*. Fifty-five samples showed resistance to colistin out of 241 samples. In colistin resistant bacteria, two *E.coli* were MBL, ESBLs, while one *E.coli* was ESBLs, AmpC co-producing bacteria. The most prevalent extended drug resistant bacteria were *Pseudomonas aeruginosa (28.6%) and Escherichia coli (*6.1%), While 155(86.6%) *Escherichia coli*, 25 (59.5%) *Pseudomonas aeruginosa* and 22 (95.7%) *Serratia Oderifora* was multi drug resistant bacteria.

**Conclusion:**

Current study concluded that ESBL, MBL AmpC enzymes and their co-expression was observed with colistin resistance in *E.coli* and *Pseudomonas aeruginosa*.

## INTRODUCTION

Worldwide approximately 150 million people are affected by urinary tract infection each year.^1^ UTIs are a major cause of morbidity in children, elderly men, and women of all ages.^2^ Both gram negative and gram-positive bacteria, as well as certain fungi, can cause UTIs. The most common causative agent for UTIs is uropathogenic *Escherichia coli* (UPEC).^3^ *Klebsiella pneumoniae, Pseudomonas aeruginosa, Proteus mirabilis, Enterococcus faecalis, group B Streptococcus (GBS), Staphylococcus aureus and Candida spp* are followed *by E.coli* for causing UTI.^4^

Development of different antibiotic resistance mechanisms and widespread emergence by uropathogenic bacteria, UTIs are difficult to treat.^5^ Enterobacteriaceae family members, such *as E. coli and K. pneumoniae*, have both intrinsic and acquired plasmids encoding ESBLs genes, which are of special concern.^6^ These plasmids rapidly spread resistance to third generation cephalosporins as well as other antibiotics.^5^ Other Enterobacteriaceae family members produce the class C βlactamases (AmpC enzymes) that are active against cephamycin in addition to third generation cephalosporins, and are also resistant to β-lactamase inhibitors.^7^ The expression of AmpC enzymes is also associated with carbapenem resistance in *K. pneumoniae* strains lacking a 42 kDa outer membrane protein.^8^

Treating Multi drug resistant bacteria, colistin was used as a last line of antibiotic for gram negative bacteria.^9^ After the detection of mcr genes on plasmid of gram-negative bacteria disclosed that colistin resistance can be horizontally transferred.^10^,^11^ ESBL, MBL, AmpC producing gram negative bacteria with mcr genes harboring by Enterobacteriaceae have been discovered all over the world, putting people at risk of having no effective antibacterial treatments.^12^

The production of extended beta-lactamases (ESBL), metallo beta-lactamases (MBL) and AmpC enzyme combined with a colistin resistance in gram-negative bacteria has a great threat to economic burden of these infections. For effective treatment against MDR uropathogenic gram negative bacteria it is important that proper and continuous surveillance of antimicrobial resistance pattern with ESBL, MBL, AmpC and colistin resistance should be done in Pakistan. That’s why this study was designed to find the ESBL, MBL and AmpC in colistin acquired and intrinsically resistant in uropathogenic Gram Negative bacteria.

## METHODS

Urine samples for this cross-sectional study was collected from Hayatabad Medical Complex, Peshawar during January to June 2019. Non probability sampling technique was applied for sampling procedure.

### Ethical Approval

It was obtained from the institutional ethical review board (IREB) of HMC Peshawar with reference number.144/HEC/B&PSC/19, dated: 16 January 2019. After taking permission from the hospital, the ethical clearance was obtained from ethical committee of Khyber Medical University Peshawar. Mid-stream urine samples were collected from infected patients in sterile urine collection bottles. All samples were aseptically transported to microbiology laboratory of Health Research Institution, Research Centre (NIH), KMC, Peshawar. Gram positive bacteria and fungi were excluded from present study.

### Procedure

Urine samples were streaked on Nutrient agar (Oxoid Limited, UK), MacConkey agar (Oxoid Limited, UK), EMB agar (Oxoid Limited, UK), CLED with android indicator (Oxoid Limited, UK) media and incubate it under aerobic condition at 37ºC for 24 hours in incubator. Gram negative bacteria was identified through Gram staining. The gram-negative bacteria were conformed through API-10s system according to kit procedure (bioMérieux, France). Modified Kirby Bauer disc diffusion method was used for antibiotic sensitivity and resistance testing. The bacterial growth was adjusted in sterile saline water to 0.5 McFarland standard solutions and streaked on Muller Hinton agar for antibiogram. Results were interpreted according CLSI guideline.^3^,^13^ The identified samples were divided in two categories acquired and intrinsic colistin resistant bacteria.^14^ Colistin resistance of acquired bacteria was identified by broth microdilution according to the CLSI guideline.^3^ MDR, XDR, and PDR uropathogenic strains were identified as per criteria defined by CDC and ECDC.^15^

Phenotypic detection of metallo βlactamases production by Imipenem EDTA synergy test: For the detection of MBL production bacterial growth was adjusted to 0.5 McFarland standard solutions and streaked on Muller Hinton agar with help of sterile swab. Place two Imipenem disc on the MHA plate and 4 µl of 0.5M EDTA solution was added to one of them to obtain the desired concentration (750 μg). Incubate the plate at 37ºC for 24 hours. After overnight incubation, the inhibition zone of Imipenem and Imipenem EDTA discs was compared. The increase in inhibition zone with Imipenem EDTA disc is ≥5mm than the Imipenem disc alone, was considered as MBL positive.^6^

### Double Disc Synergy Test (DDST) for detection of ESBLs

For phenotypic detection of ESBLs, in sterile saline water bacterial suspension was adjusted to 0.5 McFarland solution. The inoculated bacterial culture was streaked with help of sterile swab on MHA media. On inoculated plate amoxicillin/clavulanic acid ((20 μg amoxicillin+10 μg clavulanic acid) disc was placed in center. Aztreonam, cefepime, cefotaxime, ceftriaxone, imipenem, and ceftazidime were placed around the amoxicillin/clavulanic acid disc). Incubate it at 37ºC for 24 hours. Zone of inhibition toward amoxicillin/clavulanic acid disc or ghost inhibition zone appears between the central disc and any of the other antibiotics was considered as ESBLs positive.^6^

### D-test for the detection of inducible AmpC beta lactamases

Bacterial suspension was adjusted according to the 0.5 McFarland solution and streaked on the MHA with sterile swab. Imipenem disc was placed in center and place the ceftazidime and piperacillin/tazobactam on each side. Incubate the plate at 37ºC for 24 hours. AmpC production was considered positive if the D-shaped inhibition zone is observed for one of the disc (ceftazidime or piperacillin/tazobactam)^6^.

### Statistical analysis

Collected data was entered in MS- Excel sheet and data were analyzed in SPSS version 20(IBM corporation). Qualitative data was analyzed in percentage and frequency.

## RESULTS

During the sampling period total 2000 urine samples were collected from symptomatic patients of UTI. Among them 281(14%) gram-negative bacteria were isolated from urine. Among 281 positive samples, 156 (55.5%) female and 125 (44.5%) males were infected with uropathogenic gram negative bacteria. The leading uropathogenic bacteria was *E.coli* 179(63.7%) followed by other gram negative bacteria (Table-I). The gram-negative bacteria were divided in two groups on the basis of colistin resistance e.g. intrinsically resistant bacteria and acquired resistant bacteria.

**Table-I:** Complete profile of intrinsically and colistin resistant bacteria isolated from UTI patients.

Bacterial isolates	Total number of isolates (281)	Sensitive to colistin bacterial isolates N (%)	Acquired resistance N (%)	intrinsically resistant N (%)	ESBL producing bacteria number N (%)		MBL N (%)		AmpC N (%)	

					NEG	POS	NEG	POS	NEG	POS
Elizabethkingia meningoseptica	1	NA	NA	1(100)	1(100)	0	1(100)	0	0	1(100)
Enterobacter aerogenes	4	4	0	NA	4(100)	0	3(75)	1(25)	3(75)	1(25)
Enterobacter Clocaea	2	2(100)	0	NA	2(100)	0	2(100)	0	2(100)	0
Escherichia coli	179	131(73.2)	48(26.8)	NA	124(69.3)	55(30.7)	158(88.3)	21(11.7)	165(92.2)	14(7.8)
Klebsiella oxytica	2	2	0	NA	2(100)	0	2(100)	0	2(100)	0
Klebsiella pneumoniae	11	6(60)	4(40)	NA	8(80)	2(20)	8(80)	2(20)	11(100)	0
Pantoea spp	1	NA	NA	1(100)	1(100)	0	1(100)	0	1(100)	0
proteus marblis	8	NA	NA	8(100)	6(75)	2(25)	8(100)	0	7(87.5)	1(12.5)
proteus spp	2	NA	NA	2(100)	1(50)	1(50)	2(100)	0	2(100)	0
Pseudomonas aeruginosa	42	39(92)	3(7.1)	NA	35(83.3)	7(16.7)	25(59.5)	17(40.5)	30(71.4)	12(28.6)
Serratia MARCESCNS	5	NA	NA	5(100)	3(60)	2(40)	4(80)	1(20)	5(100)	0
Serratia ODERIFORA	23	NA	NA	23(100)	18(78.3)	5(21.7)	21(91.3)	2(8.7)	23(100)	0
Shigella spp.	1	1	0	NA	1(100)	0	1(100)	0	1(100)	0

NA: Not Applicable NEG: negative POS: positive

Among 281 samples, 241 samples were identified for having ability to acquired colistin resistance and 40 samples were intrinsic resistant bacteria. Among 241 samples, 55 samples were colistin resistant bacteria identified by broth microdilution and 186 samples were sensitive to colistin as shown in Table-I. Table-I indicates that 21 (11.7%) *E.coli* and 17 (40.5%) *Pseudomonas aeruginosa* were Metallo beta lactamases producing bacteria. Among ESBL producing bacteria, the most common was *E. coli, Pseudomonas aeruginosa, Klebsiella Pneumoniae, Serratia Oderifora and Proteus Marblis* isolated during the study. AmpC production was observed mostly in *E. coli* 14 (7.8%) and *Pseudomonas aeruginosa* 12 (28.6%).

**Fig.1:**
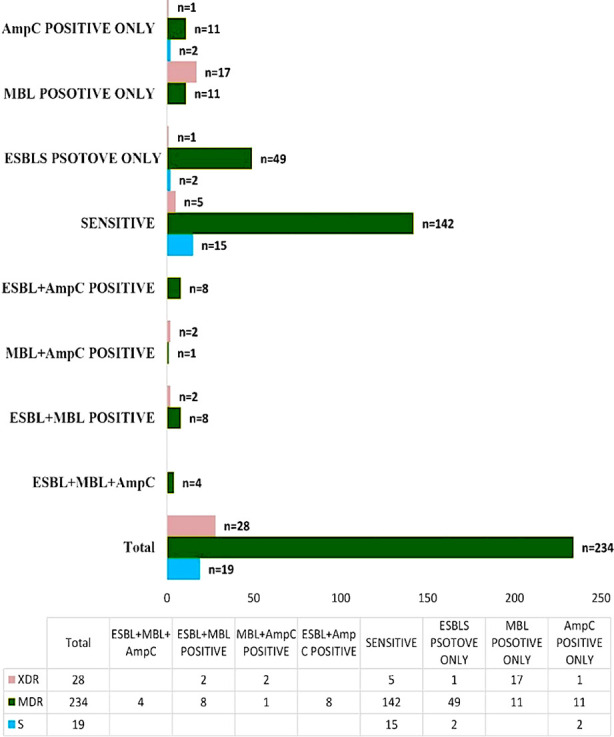
Co-existence β-Lactamases Production in Sensitive, MDR and XDR bacteria.

Table-II shows that 12(28.6%) *Pseudomonas aeruginosa and 11(6.1%) Escherichia coli* were XDR bacteria isolated from UTI patients. While 155(86.6%) *Escherichia coli*, 25(59.5%) *Pseudomonas aeruginosa* and 22 (95.7%) *Serratia Oderifora* were MDR bacteria. ESBL, MBL and AmpC were co-expressed in four MDR uropathogenic bacteria. ESBL, MBL co-existence was found in eight MDR bacteria and two XDR bacteria. Eight MDR bacteria were producing ESBL and AmpC. MBL and AmpC were expressed in one MDR and two XDR bacteria (Fig.1).

**Table-II:** Categories of resistance from isolated uropathogenic bacteria.

Bacteria	Total number species	Sensitivity and resistance pattern		

		Sensitive	MDR	XDR
Elizabethkingia meningoseptica	1	0	1(100%)	0
Enterobacter aerogenes	4	0	3(75%)	1(25%)
ENTEROBACTER CLOCAEA	2	0	2(100%)	0
Escherichia coli	179	13(7.3%)	155(86.6%)	11(6.1%)
Klebsiella oxytica	2	0	2(100%)	0
Klebsiella pneumoniae	11	1(9.1%)	8(81.8%)	1(9.1%)
Pantoea spp	1	0	1(100%)	0
proteus marblis	8	0	7(87.5%)	1(12.5%)
proteus spp	2	0	2(100%)	0
Pseudomonas aeruginosa	42	5(11.9%)	25(59.5%)	12(28.6%)
Serratia marcescns	5	0	4(80%)	1(20%)
Serratia oderifora	23	0	22(95.7%)	1(4.3%)
Shigella spp	1	0	1(100%)	0

Spp: specie MDR: Multi drug resistance XDR: Extended drug-resistance.

**Fig.2:**
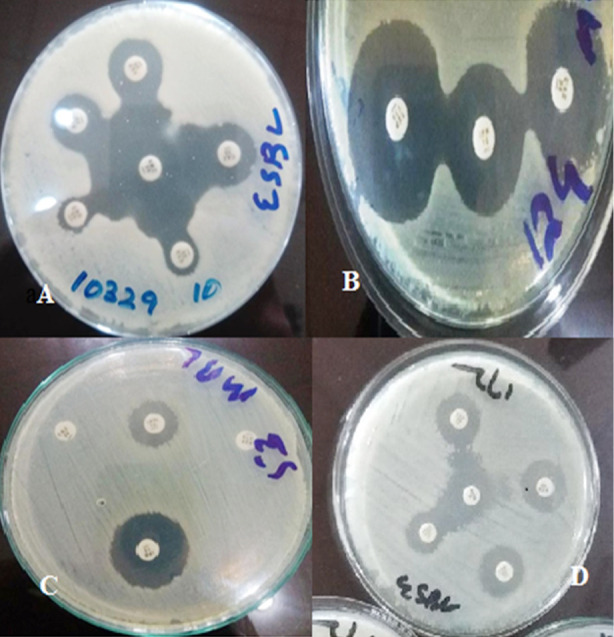
A: ESBL producing bacteria, B: AmpC producing bacteria, C: MBL producing bacteria, D: ESBL producing bacteria.

Three *Pseudomonas aeruginosa* isolates and one *Escherichia coli* showed the co-existence of ESBL.Table-III. MBL and AmpC. ESBL and MBL were co-produced by five colistin sensitive *E. coli*, two colistin resistant *E.coli*, one in intrinsically colistin resistant *Serratia marcescns* and *one in Serratia oderifora* which is also intrinsically resistant colistin. MBL and AmpC co-production were observed in three colistin sensitive *Pseudomonas aeruginosa*. In colistin sensitive bacteria, the AmpC and ESBL co-existence were found in five *E. coli, one Pseudomonas aeruginosa*, one in colistin resistant *E.coli*, and one in intrinsically resistant colistin *Proteus marbles*.

**Table-III:** Co-existence β-Lactamases Production in isolated uropathogenic bacteria.

	ESBL+ MBL+ AmpC	ESBL+MBL	MBL+ AmpC	ESBL+ AmpC	Sensitive	ESBL only	MBL only	AmpC only
***Colistin Sensitive Bacteria***								
Enterobacter aerogenes	0	0	0	0	2	0	1	1
Enterobacter Cloacae	0	0	0	0	2	0	0	0
Escherichia coli	1	5	0	5	78	29	8	5
Klebsiella oxytica	0	0	0	0	2	0	0	0
Klebsiella pneumoniae	0	1	0	0	5	0	1	0
Pseudomonas aeruginosa	3	0	3	1	13	3	11	5
Shigella spp.	0	0	0	0	1	0	0	0
***Colistin resistant bacteria***								
Escherichia coli	0	2	0	1	26	12	5	2
Klebsiella pneumoniae	0	0	0	0	2	1	1	0
Pseudomonas aeruginosa	0	0	0	0	3	0	0	0
***Intrinsically resistant bacteria***								
Elizabethkingia meningoseptica	0	0	0	0	0	0	0	1
Pantoea spp	0	0	0	0	1	0	0	0
proteus marblis	0	0	0	1	6	1	0	0
proteus spp	0	0	0	0	1	1	0	0
Serratia marcescns	0	1	0	0	3	1	0	0
Serratia oderifora	0	1	0	0	17	4	1	0

## DISCUSSION

According to present study the prevalence of UTI associated with gram negative bacteria was 14.05%. A study done in Pakistan showed that 30% prevalence of UTI associated with gram negative among suspected patients.^16^ In another study in Pakistan showed that prevalence of UTI associated with gram negative bacteria was 5.3%.^17^ In present study female were more infected then male, the same results were also documented by other studies.^17^,^16^ The leading cause of UTI in this study was *E.coli* (63.7%). The same results were also observed in other studies.^16^-^18^

This study results were further divided into intrinsic and acquired resistant gram-negative bacteria. *Burkholderia cepacia complex, Providencia spp, Serratia spp, Edwardsiella tarda, Proteus spp and Morganella morganii* are intrinsically resistant gram negative bacteria.^19^ In present study *Elizabethkingia meningoseptica, Pantoea spp, proteus marblis, Serratia marcescns, Serratia oderifora* were isolated from UTI patients. *Elizabethkingia meningoseptica* and *Shigella spp* were also isolated from UTI patients from other studies.^20^,^21^

*E.coli, K. pneumoniae, Pseudomonas aeruginosa, Enterobacter, Salmonella, Shigella and Acinetobacter baumannii* are the most common acquired resistant gram-negative bacteria to colistin antibiotic.^22^ According to the present data colistin resistant bacteria such as *E.coli, K. pneumoniae and Pseudomonas aeruginosa* were isolated.

The presence Metallo beta lactamases in 21(11.7%) *E.coli* and *17(40.5%) Pseudomonas aeruginosa* were identified in this study. A research conducted in Swabi Pakistan showed that 12 (16%) E.*coli* isolates were MBL producing uropathogenic bacteria.^23^ A study done in Nepal showed that 14 uropathogenic gram negative isolates were showed the production of MBL, among which 5 (35.71%) *E.coli* and 4 (28.57%) *Pseudomonas aeruginosa* were MBL producer.^24^ Another study results showed that 8% *E.coli and 17% K. pneumoniae* were MBL producing bacteria.^6^

According to this research 74 (26.3%) gram negative isolates showed ESBL production, among them most common were *E. coli, Pseudomonas aeruginosa, Klebsiella Pneumoniae, Serratia Oderifora and Proteus Marblis*. These results are also comparable to the other studies done in Nepal.^6^,^24^ Another study done in Pakistan showed that 25(33.3%) uropathogenic *E coli* isolates were producing ESBL.^23^

In a current research AmpC enzyme production was observed in 14 (7.8%) *E. coli* and 12 (28.6%) *Pseudomonas aeruginosa*. A study completed in Iran disclosed that AmpC enzyme was detected in 29 (61.7%) uropathogenic *E coli*.^25^ A study completed in India showed that 25(11.7%) E. coli and 11(17%) *Klebsiella Pneumoniae* was AmpC producing bacteria.^6^ ESBL, MBL and AmpC enzymes were co-expressed in four uropathogenic gram negative bacteria, predominantly in *Pseudomonas aeruginosa* (n=3). But the studies carried out in India showed that co-expression of ESBL, MBL and AmpC enzymes were most commonly in *E. coli^26^ and Klebsiella Pneumoniae*.^6^ The co-production of ESBL and MBL were mostly observed in *E. coli* bacteria in present study.

The prevalence of ESBL and MBL in combination production in other study was much higher in *E. coli* then present study.^27^ MBL+ AmpC co-production were detected mostly in *Pseudomonas aeruginosa* (n=3) in current study. In recent study of Iran showed that the presence of both MBL+ AmpC enzymes were in 18 (38.3%) uropathogenic *E. coli*.^25^ The co-expression of ESBL and AmpC enzymes were found in eight Enterobacteriaceae samples in this study. While study done in Sri Lanka reported much higher rate of ESBL and AmpC prevalence then present study.^28^

Twelve colistin resistant *E.coli* and one colistin resistant *K. pneumoniae* were able to produce the ESBL enzymes according to this study finding. Finding of this study also revealed that five colistin resistant *E. coli* isolates and one colistin resistant *K. pneumoniae* were MBL producing bacteria. Two *E.coli* isolates were having ability to produce AmpC enzymes. A study completed in Nigeria finding showed that five colistin resistant *K.pneumoniae* produced ESBL and AmpC and three produced MBL enzymes.^29^

In our study two isolates of colistin resistant *E. coli* were able to produce the ESBL and MBL concurrently. While another study result was different from current study showed that, four ESBL and AmpC, three ESBL and MBL, two AmpC and MBL co-production were found in colistin resistant *K. pneumoniae*. The co expression of ESBL, MBL and AmpC found in three colistin resistant *K. pneumoniae* isolates.^29^ Co-secretion of these beta- lactamases enzymes in current study provides the idea of horizontal transfer of related resistance genes among same and different isolates. These results emphasize on continuous surveillance of XDR and MDR uropathogenic bacteria for appropriate therapy.

### Limitations

This study was limited to only UTI patients and conducted in one hospital of Peshawar. It might expand our information about the causative bacterial agent pattern of UTI, antibiotic resistant profile and most essential colistin resistance with other beta lactamase enzymes production, if samples are selected from different hospitals and other infections. Moreover, current study was only limited to phenotypic identification. Similar studies are required on molecular level to understand the possible mechanisms responsible for causing colistin resistance and production of different beta lactamases.

## CONCLUSION

The co-production of MBL, ESBL and Ampc enzymes with colistin resistance in uropathogenic bacteria in this study is great alarming situation for the treatment of UTI patients. *E. coli* and *Pseudomonas aeruginosa* was the leading bacteria in production of MBL, ESBL and AmpC enzymes co-currently. Higher number of multidrug resistant gram negative bacteria was isolated from UTI patients in this study, which further narrow down the treatment options.

### Author’s Contribution:

**AA:** Conceived, designed, did manuscript writing.

**IU:** Did review and final approval of manuscript, responsible for integrity of research.

**RZ:** Did statistical analysis & editing of manuscript

**AMK:** Did data collection and editing of manuscript.
